# Anxiety is not enough to drive me away: A latent profile analysis on math anxiety and math motivation

**DOI:** 10.1371/journal.pone.0192072

**Published:** 2018-02-14

**Authors:** Zhe Wang, Nicholas Shakeshaft, Kerry Schofield, Margherita Malanchini

**Affiliations:** 1 Department of Human Development and Family Studies, Texas Tech University, Lubbock, TX, United States of America; 2 MRC Social, Genetic, and Developmental Psychiatry Centre, King’s College London, London, United Kingdom; 3 Department of Psychology, University of Texas at Austin, Austin, TX, United States of America; University of Zurich, SWITZERLAND

## Abstract

Mathematics anxiety (MA) and mathematics motivation (MM) are important multi-dimensional non-cognitive factors in mathematics learning. While the negative relation between global MA and MM is well replicated, the relations between specific dimensions of MA and MM are largely unexplored. The present study utilized latent profile analysis to explore profiles of various aspects of MA (including learning MA and exam MA) and MM (including importance, self-perceived ability, and interest), to provide a more holistic understanding of the math-specific emotion and motivation experiences. In a sample of 927 high school students (13–21 years old), we found 8 distinct profiles characterized by various combinations of dimensions of MA and MM, revealing the complexity in the math-specific emotion-motivation relation beyond a single negative correlation. Further, these profiles differed on mathematics learning behaviors and mathematics achievement. For example, the highest achieving students reported modest exam MA and high MM, whereas the most engaged students were characterized by a combination of high exam MA and high MM. These results call for the need to move beyond linear relations among global constructs to address the complexity in the emotion-motivation-cognition interplay in mathematics learning, and highlight the importance of customized intervention for these heterogeneous groups.

## Introduction

Mathematics anxiety (MA) and mathematics motivation (MM) are important multi-faceted non-cognitive factors in mathematics learning. MA refers to the fear and apprehension experienced prior to or during math-related activities [1]. MM captures the extent to which individuals value the importance of math abilities, are interested in math-related activities, and are motivated to perform well in math [2]. Although studies consistently reported modest to moderate negative correlations between MA and MM [3, 4], their relations are likely more intricate than a negative linear association.

Wang and colleagues [5] argued that MA and MM are conceptually related but distinctive. They are related because both capture the valence dimension of math-related experiences, with MA capturing the negative evaluation (e.g., fear and uneasiness) and MM the positive evaluation (e.g., interest and reward). Yet, MA and MM are distinct constructs rather than two opposing ends of a continuum. MM captures the motivation dimension which defines the approach versus withdrawal orientation toward math activities, whereas MA offers little information in this regard. In other words, students experiencing apprehension about math activities may avoid similar situations in the future [6], or they may overcome such emotional challenges by investing more effort [7, 8], with these differential responses being likely related to how motivated they are. This conceptualization is consistent with factor analytic evidence showing that MA and MM are two separate but modestly correlated constructs [9, 10]. Such a conceptualization points to an intricate multi-dimensional emotion-motivation relation that requires further investigation.

Additionally, both MA and MM are multi-faceted constructs. Depending on the instruments used to measure MA, different factor structures were found. The four most common factors are anxiety about math tests [11–13], anxiety about performing numerical operations [11–12, 14], anxiety about performing math in social situations [12–13], and anxiety about observing and learning materials in math [3, 15]. With respect to motivation, the three most studied dimensions are self-perceived ability, interest, and importance [2]. Self-perceived ability measures individuals’ perception of their competence in various math tasks. Interest indicates the enjoyment one gains from learning and doing math. Importance refers to the perceived importance of doing well in math. Given that both constructs are multi-faceted, it is possible that different aspects of MA and MM relate to one another in distinct ways. For example, students who dread learning new materials in math are unlikely to enjoy math learning, but they may still consider it important to master math. Students who feel competent in their math ability may still worry about making a mistake in an upcoming exam. Therefore, a single correlation between global MA and MM seems insufficient in capturing these complex multi-dimensional relations. To address this gap, the first aim of the current research is to examine the relations among the specific dimensions of MA and MM.

The ultimate goal in examining emotion and motivation experiences is to understand how they relate to math learning and achievement. Many existing studies examined how MA and MM were each associated with math achievement and learning behaviors. Higher MM and lower MA were, respectively, associated with higher math achievement [4, 16–19], and with more engagement in math-related activities such as taking more elective math courses [4, 6, 20]. However, few studies to date examined the combined roles of MA and MM and the possibility of their interactive and nonlinear effects on mathematics learning. One study showed that after accounting for MM, MA is no longer associated with intention to select math courses [4], suggesting that math avoidance is primarily associated with the motivation dimension. Two recent studies revealed that MM moderates the relation between MA and math performance [5, 7], such that high MM mitigates the negative association between MA and math performance. Together, these studies suggested that understanding the complex relations between emotion, motivation and cognition in mathematics requires investigating diverse noncognitive characteristics in conjunction rather than in isolation. Therefore, the second aim of the present study was to explore differences in math achievement and math learning avoidance among students with different emotional and motivational profiles.

Given that so few studies have examined the relations among specific dimensions of MA and MM, the current study took an explorative approach by using latent profile analysis to further our understanding of the emotion and motivation experiences in mathematics learning. Latent profile analysis is a more holistic approach than variable-centered approaches (for example moderation analysis) in studying relations that are multidimensional in nature, as it allows the discovery of heterogeneous groups of individuals with similar values on the multiple dimensions of interest. The different profiles derived from latent profile analysis represent naturally occurring groups of individuals in the population, characterized by distinctive combinations of various math-related emotional and motivational experiences. A latent profile analysis approach also allows for the examination of the differences in math achievement and math avoidance among students with different emotional and motivational profiles. It would be extremely difficult to study how the multiple dimensions of MA and MM work together in relation to achievement and avoidance using a variable-centered approach, especially if taking into consideration both linear and curvilinear, as well as additive and interactive effects. The advantage of latent profile analysis is that it narrows our focus on the naturally occurring combinations of various dimensions of MA and MM in the population, as opposed to artificially dividing the sample into arbitrary categories. By comparing the means of math achievement and avoidance across these profiles, this approach allows us to explore how the existing combinations of MA and MM profiles relate to math achievement and avoidance without assuming linearity and additivity in their relations.

## Methods

### Participants

This work is part of the Multi-Cohort Investigation into Learning and Educational Success (MILES) study. MILES is an accelerated longitudinal study which aims to investigate the factors contributing to individual differences in academic achievement and psychological wellbeing over the course of high school in Italy. All students from three opportunistically-selected high schools in the Province of Milan were invited, and 1020 participated in the first wave of data collection in March 2016. After data cleaning and screening, 927 students (437 male, 490 female) contributed data to the present investigation. The age of the students ranged from 13 to 21 years (*M* = 15.87, *SD* = 1.49).

### Procedure

All students had been previously informed about the aims and procedures through conferences held in the schools by the MILES team. The data were collected online via the MILES website (www.projectmiles.com/test), using the forepsyte.com online platform (www.forepsyte.com). The first wave of data collection lasted around 90 minutes and included cognitive tests and self-report measures.

MILES received ethical approval from Goldsmiths University of London. The parents’ and teachers’ committees of every school approved the MILES project and data collection protocol. Approval was received prior data collection, and is renewed every year. Every student was presented with an online information sheet explaining the motivation of the research conducted and completed an online consent form in Italian. Each student was informed that participation was voluntary and that they could withdraw from the study at any time. Data will be made available to researchers upon request and completion of the MILES research collaboration form (http://www.projectmiles.com/research.html).

### Measures

All measures were translated to and administered in Italian. All translated measures were piloted on a sample of 70 students from five high schools in the Province of Milan prior to the first wave of data collection. The factor structure, distribution of constructs, and the associations between constructs were comparable to those obtained with the validated measures administered to English-speaking samples.

#### Mathematics motivation (MM)

Three aspects of MM were assessed. The 1^st^ aspect was attitude towards mathematics (i.e., importance) which was measured using 1 item retrieved from the PISA study (OECD Program for international student assessment, www.pisa.oecd.org). Students were asked to rate on a 4-point scale “how important do you think it is to do well in mathematics” (1 = not at all; 4 = very much). For the ease of comparison with other scales, Importance was rescaled to a 1–5 scale using min-max normalization. Results remained the same regardless of the transformation.

The 2^nd^ aspect of motivation was self-perceived ability in mathematics (i.e., self-perception). Students were asked to rate how good they thought they were at specific math activities on a 5-point scale (1 = not good at all; 5 = very good) [21]. Specific abilities included solving number and money problems, doing math in their head, and multiplying and dividing. Cronbach’s alpha for this scale was 0.77.

The 3^rd^ aspect of motivation was enjoyment of mathematics (i.e., interest). Students were asked to rate how much they enjoyed the above 3 activities on a 5-point scale (1 = not like it at all; 5 = like it very much) [21]. Cronbach’s alpha for this scale was 0.79.

#### Mathematics anxiety (MA)

MA was measured using The Abbreviated Math Anxiety Scale (AMAS) [22]. Students were asked to rate on a 5-point scale how anxious/nervous they felt in several math-related contexts and activities (1 = not all all; 5 = very much). Principal component analysis with oblimin rotation showed a clear 2-component structure, with the two components explaining 46% and 15% of the total variance, respectively. Five items loaded on the 1^st^ component which captured anxiety about learning new math materials or listening to others’ explaining math (loadings ranged from 0.55 to 0.86). Three items loaded on the 2^nd^ component which captured anxiety about math exams (loadings ranged from 0.84 to 0.90). One item had double loadings and was excluded from the analysis to avoid contamination between components. We labeled the 2 components *learning MA* and *exam MA*. Both subscales were internally consistent with Cronbach’s alphas of 0.79 and 0.87, respectively. Higher scores indicated higher MA.

#### Mathematics achievement

Students self-reported their grades in mathematics from the semester that had ended in January, prior to the collection wave in February-March 2016. Scores ranged from 4 (indicating a grade equivalent to 4 or less than 4) to 10 (indicating the highest possible grade), with 6 indicating the pass mark.

#### Mathematics time

Students were asked to rate how much time they spent on “Out of school time lessons in mathematics” and “Study for homework in mathematics myself” on a 5-point scale (1 = no time; 2 = less than 2 hours; 3 = 2 to 4 hours; 4 = 4 to 6 hours; 5 = 6 or more hours; OECD Program for international student assessment, www.pisa.oecd.org). The two items were modestly correlated (*r* = 0.32), and were averaged to obtain a single score representing time spent on mathematics after school. A higher score represents more time spent on afterschool math learning and less avoidance.

### Analytic strategies

All analyses were conducted in SPSS Version 24 [23] and Mplus Version 7.4 [24]. First, descriptive and correlational analyses were conducted to understand the basic properties of the variables. Next, latent profile analysis (LPA) was performed on the multidimensional aspects of MM and MA: math importance, self-perceived ability in math, interests in math, learning MA, and exam MA. The best model was selected using Bayesian Information Criterion (BIC) [25] as the primary criterion, and Lo-Mendell-Rubin adjusted likelihood ratio test (LMRT) [26] as the supplementary criterion, in order to establish the number of classes and to examine the distinct features of these classes. Next, we examined whether sex and grade level predicted class memberships using the r3step command in Mplus. Subsequently, we examined whether students in different classes also differed on math achievement and math time using ANOVA.

## Results

### Descriptive and correlational analyses

Descriptive statistics are shown in Table 1. All variables were distributed widely across their entire scales. The mean was lower for learning MA compared to exam MA, suggesting that exam MA was more prevalent compared to learning MA.

**Table 1:** Descriptive statistics of Main Study Variables.

	N	Mean	Std. Dev	Skewness	Kurtosis	Median	Min	Max
Importance	927	3.74	1.12	-0.44	-0.70	3.67	1.00	5.00
Self-perception	927	3.53	0.80	-0.69	0.57	3.67	1.00	5.00
Interest	927	3.03	1.00	-0.26	-0.49	3.00	1.00	5.00
Learning MA	927	1.75	0.73	1.31	1.65	1.60	1.00	5.00
Exam MA	927	3.61	1.07	-0.58	-0.56	3.67	1.00	5.00
Achievement	927	6.53	1.40	0.01	-0.55	7.00	4.00	10.00
Time	927	2.25	0.69	1.12	1.89	2.00	1.00	5.00

Note. MA = math anxiety.

Correlations are shown in Table 2. Female students reported higher MA and lower MM compared to male students. Grade levels were weakly negatively associated with importance, suggesting that students in higher grade levels tended to view math as less important. Exam MA and learning MA were moderately positively correlated, and so were different aspects of MM. Both exam MA and learning MA were modestly negatively correlated with various aspects of MM. Math achievement was associated modestly negatively with MA and modestly positively with MM. Math time was positively associated with exam MA, importance, and interest. Finally, math time was modestly negatively associated with math achievement.

**Table 2:** Correlations between Main Study Variables.

	1	2	3	4	5	6	7	8
1. Sex	—							
2. Grade	-0.01	—						
3. Importance	-0.14*	-0.11*	—					
4. Self perception	-0.31*	-0.07	0.39*	—				
5. Interest	-0.19*	-0.05	0.44*	0.67*	—			
6. Learning MA	0.19*	0.05	-0.29*	-0.41*	-0.33*	—		
7. Exam MA	0.26*	-0.05	-0.20*	-0.37*	-0.33*	0.47*	—	
8. Achievement	0.06	0.04	0.25*	0.29*	0.22*	-0.21*	-0.27*	—
9. Time	0.07	0.02	0.12*	0.03	0.11*	0.08	0.22*	-0.11*

Note. MA = math anxiety. Pre-specified Type I error rate is 0.05

*indicates statistical significance after Holm-Bonferroni correction.

### Latent profile analysis

Latent profile analysis was performed to explore profiles of MA and MM. Nine models from 2- to 10-Classes were run, and the best model was selected using BIC and LMRT. As shown in Table 3, BIC decreased as the number of classes increased, but the decrease became minimal beginning from the 7 class to the 8 class model. According to LMRT, the 8 class model was better than the 7 class model whereas the 9 class model was not better than the 8 class model. Therefore, the 8 class model was selected as the best model.

**Table 3:** Model fit indices for the 2- to 10- class models.

Model	Log Likelihood	Free parameters	BIC	LMRT
2 Classes	-5817.09	16	11743.49	832.95*
3 Classes	-5671.79	22	11493.89	283.67*
4 Classes	-5603.56	28	11398.42	133.21
5 Classes	-5524.60	34	11281.49	154.16
6 Classes	-5478.91	40	11231.10	89.21
7 Classes	-5439.93	46	11194.13	76.11*
8 Classes	-5408.27	52	11171.79	61.82*
9 Classes	-5382.80	58	11161.85	49.72
10 Classes	-5358.23	64	11153.70	47.97

Note. BIC = Bayesian Information Criterion. LMRT = Lo-Mendell-Rubin adjusted likelihood ratio test.

* indicates statistical significance under the pre-specified Type I error rate of 0.05.

**Fig 1:**
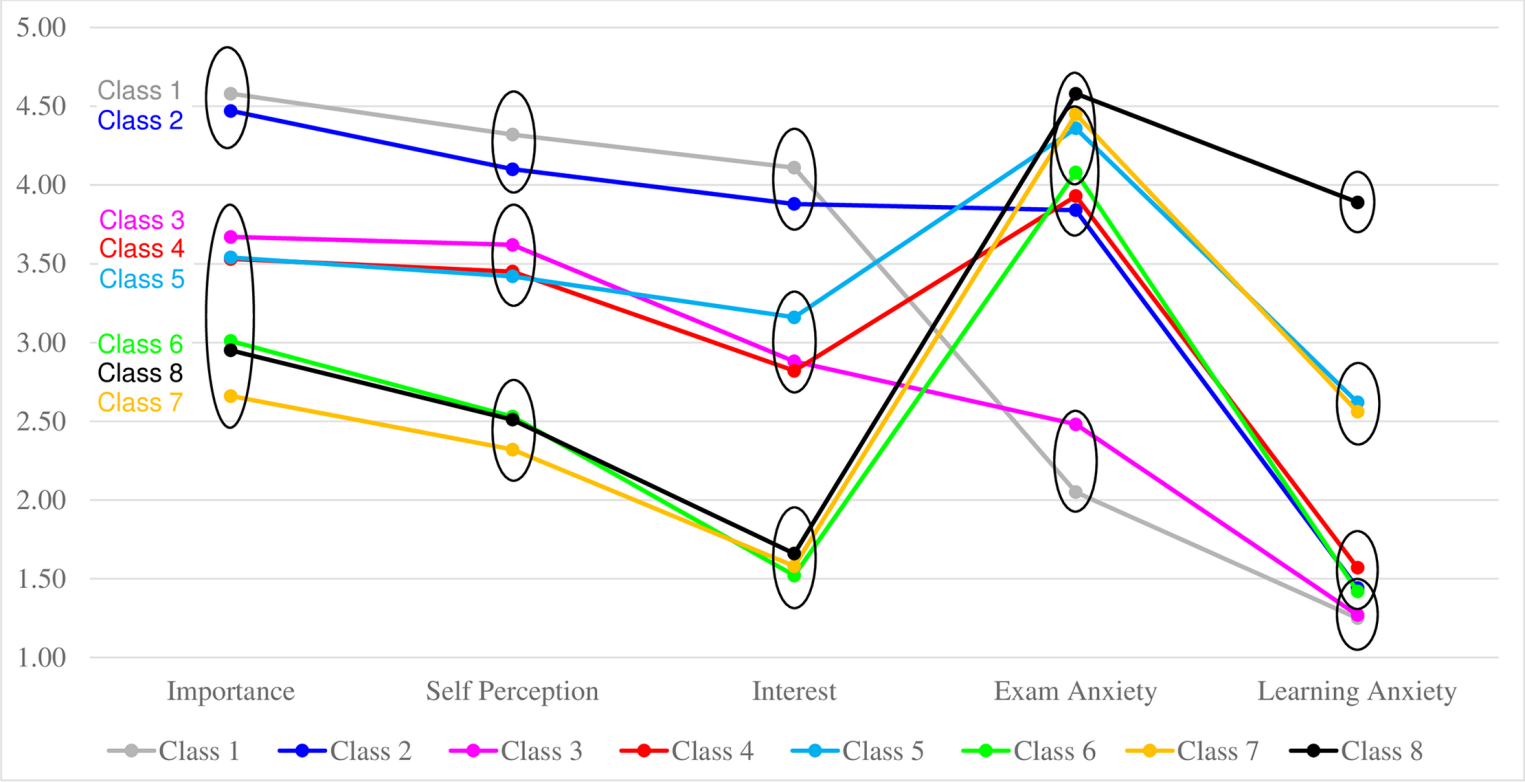
Latent profile analysis: Results from 8-class model. Means in different ellipses are significantly different from one another at the pre-specified Type I error rate of 0.05 after Bonferroni correction.

Fig 1 and Table 4 depict the characteristics of each of the 8 classes. Due to the large number of classes, here we first describe the rules we used to order and label the 8 classes: As shown in Fig 1, when considering MM levels, the 8 classes clustered into 3 groups: the first group included 2 classes that showed high MM, the second group included 3 classes showing medium MM, and the third group included another 3 classes showing low MM. This was observed across all 3 dimensions of MM (importance, self-perception, and interest). Therefore, we first ordered the classes according to levels of MM, and labeled the 1^st^, 2^nd^, and 3^rd^ groups respectively ‘high MM’ (classes 1–2), ‘medium MM’ (classes 3–5), and ‘low MM’ (classes 6–8) groups. Within each MM group, classes further differed on levels of exam MA and learning MA. Therefore, within each MM group, we further ordered the classes according to their levels of MA such that the class showing comparatively lower MA came earlier in the sequence. For example, within high MM group, one class exhibited low learning MA and low exam MA, and the other class exhibited low learning MA and high exam MA. The two classes were respectively labeled class 1 and class 2, with class 1 exhibiting lower MA compared to class 2. In the following section, in order to abbreviate the long label for each class, we use H, M, and L to represent high, medium and low levels, and use MM, EMA, and LMA to represent math motivation, exam math anxiety, and learning math anxiety.

**Table 4:** Latent profile analysis: Estimated means and 95% confidence intervals for each class.

	Importance	Self-Perception	Interest	Learning MA	Exam MA
Class 1 (n = 117) H MM, L LMA, L EMA	4.58 (4.33, 4.82) ^a^	4.32 (4.12, 4.53) ^a^	4.11 (3.84, 4.39) ^a^	1.25 (1.15, 1.35) ^a^	2.05 (1.68, 2.42) ^a^
Class 2 (n = 178) H MM, L LMA, H EMA	4.47 (4.25, 4.69) ^a^	4.10 (3.93, 4.26) ^a^	3.88 (3.59, 4.18) ^a^	1.44 (1.31, 1.57) ^a,b^	3.84 (3.51, 4.17) ^b^
Class 3 (n = 122) M MM, L LMA, L EMA	3.67 (3.17, 4.16) ^b^	3.62 (3.37, 3.88) ^b^	2.88 (2.46, 3.29) ^b^	1.27 (1.11, 1.43) ^a^	2.48 (2.04, 2.91) ^a^
Class 4 (n = 238) M MM, L LMA, H EMA	3.53 (3.17, 3.88) ^b^	3.45 (3.15, 3.74) ^b^	2.82 (2.42, 3.21) ^b^	1.57 (1.43,1.72) ^b^	3.93 (3.59, 4.28) ^b^
Class 5 (n = 122) M MM, M LMA, H EMA	3.54 (3.21, 3.88) ^b^	3.42 (3.15, 3.70) ^b^	3.16 (2.75, 3.56) ^b^	2.62 (2.41, 2.83) ^c^	4.36 (4.13, 4.59) ^b,c^
Class 6 (n = 48) L MM, L LMA, H EMA	3.01 (2.16, 3.86) ^b^	2.53 (1.99, 3.06) ^c^	1.52 (0.90, 2.14) ^c^	1.42 (1.10, 1.73) ^a,b^	4.08 (3.53, 4.63) ^b,c^
Class 7 (n = 68) L MM, M LMA, H EMA	2.66 (1.91, 3.40) ^b^	2.32 (1.76, 2.87) ^c^	1.58 (1.11, 2.05) ^c^	2.56 (2.27, 2.84) ^c^	4.45 (4.12, 4.78) ^b,c^
Class 8 (n = 34) L MM, H LMA, H EMA	2.95 (2.22, 3.68) ^b^	2.51 (1.89, 3.12) ^c^	1.66 (1.25, 2.06) ^c^	3.89 (3.57, 4.22) ^d^	4.58 (4.29, 4.87) ^c^

Note. H = high; M = medium; L = low; MM = math motivation; LMA = learning math anxiety; EMA = exam math anxiety. For each dimension, classes are not significantly different from each other if their labels contain the same letters; classes are significantly different from each other if their labels do not contain the same letters. 95% confidence intervals are calculated based on Bonferroni correction.

#### High MM classes

Class 1 (H MM, L LMA, L EMA): Approximately 13% of the sample belonged to Class 1 (n = 117). This class reported the highest MM and lowest learning MA and exam MA among all 8 classes.

Class 2 (H MM, L LMA, H EMA): 19% of the sample were in Class 2 (n = 178). Students in this class reported very high MM, very low learning MA, but high exam MA.

The similarity between Class 1 and Class 2 was that they both showed high MM. The two classes were different on how anxious they felt about math. Specifically, students in Class 2, but not in Class 1, reported high anxiety about math exams.

#### Medium MM classes

Class 3 (M MM, L LMA, L EMA): 13% of the sample were categorized in Class 3 (n = 122). This class reported medium levels of MM, and low levels of both learning MA and exam MA. This class was similar to Class 1 in that both classes reported relatively low MA compared to all other classes. This class differed from Class 1 such that they reported lower MM compared to Class 1.

Class 4 (M MM, L LMA, H EMA): about 26% of the students belonged to Class 4 (n = 238), making this the largest class. Students in this class had medium levels of MM, low learning MA, and high exam MA. Similar to Class 2, students in Class 4 were also anxious mostly about math exams but not math learning. However, students in Class 4 reported lower MM compared to those in Class 2, which crucially differentiated the two classes.

Class 5 (M MM, M LMA, H EMA): about 13% of the students were classified into this class (n = 122). This class was characterized by medium MM, medium learning MA, and high exam MA.

These three classes were similar in that they all exhibited medium levels of MM. Yet, the three classes critically differed on levels of exam MA and learning MA, with Class 3 being low on both learning MA and exam MA, Class 4 being low on learning MA but high on exam MA, and Class 5 being medium on learning MA and high on exam MA.

#### Low MM classes

Class 6 (L MM, L LMA, H EMA): 5% of the sample belonged to Class 6 (n = 48). Students in this class reported low MM, low learning MA, and high exam MA. The MA levels in this class resembled those observed in Class 2 and Class 4. What separated Class 6 from Classes 2 and 4 was its lower MM compared to the other two classes.

Class 7 (L MM, M LMA, H EMA): about 7% of all students were in Class 7 (n = 68). This class was characterized by low MM, medium learning MA, and high exam MA. This class was similar to Class 5 such that both classes reported high exam MA and medium learning MA. However, students in Class 7 had lower MM compared with students in Class 5.

Class 8 (L MM, H LMA, H EMA): approximately 4% of the sample were classified to the last class (n = 34), making this the smallest class. This class showed very low MM and very high levels of both learning MA and exam MA.

Class 6, Class 7, and Class 8 were similar in that all three classes exhibited low levels of MM and high levels of exam MA. Yet, the three classes differed on their levels of learning MA, with Class 6 being low on learning MA, Class 7 being medium, and Class 8 being high.

Table 5 summarizes the key characteristics of each class. Several interesting patterns emerged from observing the 8 classes: 1) high exam MA appeared in combination with every MM level; 2) medium learning MA appeared in combination with only medium to low MM, but not high MM; 3) high learning MA appeared in combination with only low MM, but not medium to high MM.

**Table 5:** Summarization of key characteristics of each class.

	Low EMA			High EMA		
	Low LMA	Medium LMA	High LMA	Low LMA	Medium LMA	High LMA
**High MM**	Class 1	—	—	Class 2	—	—
**Medium MM**	Class 3	—	—	Class 4	Class 5	—
**Low MM**	—	—	—	Class 6	Class 7	Class 8

Note. MM = math motivation; LMA = learning math anxiety; EMA = exam math anxiety.

Grade level and sex were examined as predictors of class memberships. Results are shown in Table 6. All effects were obtained after controlling for school differences. Overall, grade level was not a significant predictor of class membership. Sex significantly predicted class memberships, such that female students were more likely to belong to classes characterized by a combination of lower MM and higher MA compared to male students.

**Table 6:** Predictors of class membership.

		Class 1	Class 2	Class 3	Class 4	Class 5	Class 6	Class 7	Class 8
		H MM	H MM	M MM	M MM	M MM	L MM	L MM	L MM
	Reference	L LMA	L LMA	L LMA	L LMA	M LMA	L LMA	M LMA	H LMA
Predictor	Class	L EMA	H EMA	L EMA	H EMA	H EMA	H EMA	H EMA	H EMA
	Class1								
Grade level		**—**	-0.05 (0.12)	-0.10 (0.14)	-0.02 (0.11)	-0.15 (0.13)	-0.25 (0.17)	0.18 (0.14)	0.14 (0.16)
Sex		**—**	-0.02 (0.35)	-0.26 (0.42)	0.64 (0.30)	0.82 (0.34)	1.82 (0.56)*	2.05 (0.52)*	1.62 (0.52)*
	Class 2								
Grade level		**—**	**—**	-0.05 (0.13)	0.03 (0.11)	-0.10 (0.12)	-0.20 (0.16)	0.22 (0.13)	0.18 (0.15)
Sex		**—**	**—**	-0.25 (0.38)	0.66 (0.31)	0.83 (0.32)	1.84 (0.53)*	2.07 (0.50)*	1.64 (0.51)*
	Class 3								
Grade level		**—**	**—**	**—**	0.08 (0.14)	-0.05 (0.14)	-0.15 (0.18)	0.28 (0.15)	0.23 (0.17)
Sex		**—**	**—**	**—**	0.90 (0.38)	1.08 (0.37)	2.09 (0.57)*	2.31 (0.54)*	1.88 (0.54)*
	Class 4								
Grade level		**—**	**—**	**—**	**—**	-0.13 (0.12)	-0.23 (0.17)	0.21 (0.13)	0.15 (0.15)
Sex		**—**	**—**	**—**	**—**	0.18 (0.32)	1.18 (0.56)	1.41 (0.49)	0.98 (0.50)
	Class 5								
Grade level		**—**	**—**	**—**	**—**	**—**	-0.10 (0.17)	0.32 (0.15)	0.28 (0.16)
Sex		**—**	**—**	**—**	**—**	**—**	1.01 (0.54)	1.23 (0.54)	0.81 (0.52)
	Class 6								
Grade level		**—**	**—**	**—**	**—**	**—**	**—**	0.42 (0.19)	0.38 (0.20)
Sex		**—**	**—**	**—**	**—**	**—**	**—**	0.23 (0.71)	-0.20 (0.67)
	Class 7								
Grade level		**—**	**—**	**—**	**—**	**—**	**—**	**—**	-0.04 (0.18)
Sex		**—**	**—**	**—**	**—**	**—**	**—**	**—**	-0.43 (0.67)

Note. Numbers not in parentheses are parameter estimates, and numbers in parentheses are standard errors. H = high; M = medium; L = low; MM = math motivation; LMA = learning math anxiety; EMA = exam math anxiety. Pre-specified Type I error rate is 0.05

*indicates statistical significance after Holm-Bonferroni correction.

### Relations between class memberships and math achievement/time

We examined whether class memberships were associated with math achievement and math time using ANOVA. Dummy variables representing schools were entered in the models as covariates to control for school differences.

#### Association between class memberships and math achievement

**Fig 2:**
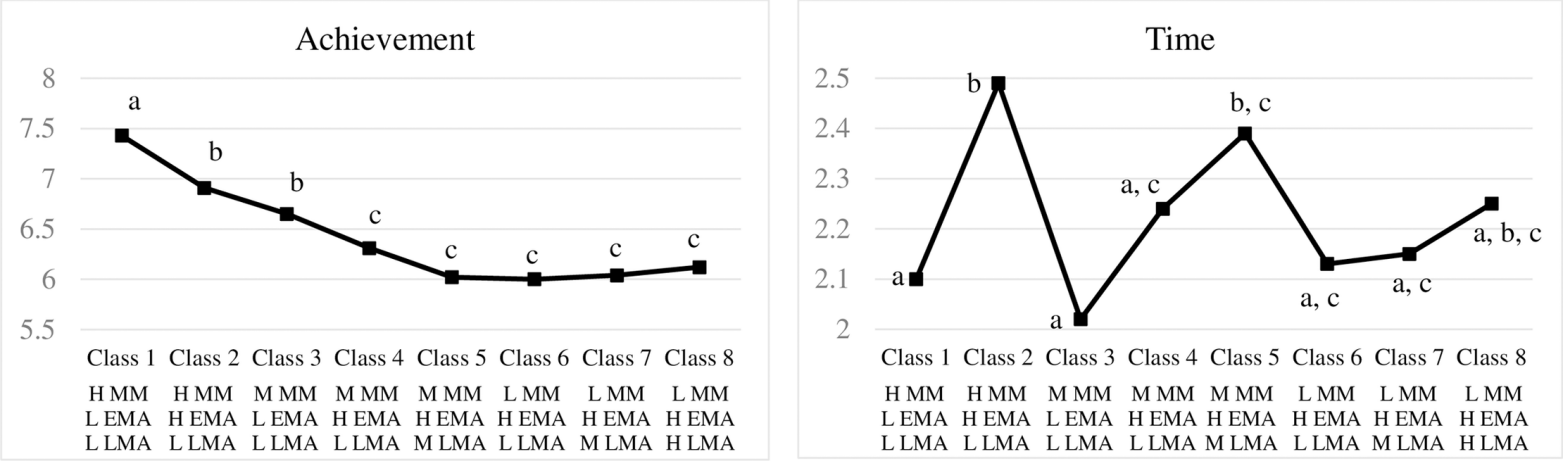
Relations between class memberships and math achievement/math time. L = low, M = medium, H = high, MM = math motivation, EMA = exam math anxiety, LMA = learning math anxiety. Classes are not significantly different from each other if their labels contain the same letters; classes are significantly different from each other if their labels do not contain the same letters. Statistical significance is calculated under the pre-specified Type I error rate of 0.05 after Holm-Bonferroni correction.

Class membership was significantly associated with math achievement, *F* (7, 917) = 20.00, *p* = 0.000, *η*^*2*^ = 0.12. Results from post-hoc contrasts are shown in the left panel in Fig 2. Class 1 performed significantly better compared to all the seven remaining classes. Class 2 and Class 3 showed similar performance that was better than that shown by Classes 4 through 8, but worse than Class 1. Finally, no significant differences in math achievement were found among Class 4 through Class 8.

#### Association between class memberships and math time

Class membership was also significantly associated with math time, *F* (7, 917) = 6.27, *p* = 0.000, *η*^*2*^ = 0.04. Results from post-hoc contrasts are shown in the right panel in Fig 2. Class 2, 5, and 8 spent the most time whereas Class 1 and 3 spent the least time studying math after school. It is worth noting that Class 2, Class 5, and Class 8 were the classes with the highest overall MA within each MM group, and they were also the classes that reported spending the most time on after-school math learning. To the contrary, Class 1 and Class 3 were the classes with the lowest overall MA within each MM group, and they were also the classes that reported spending the least amount of time on after-school math learning.

### An exploration of the developmental pattern

**Fig 3:**
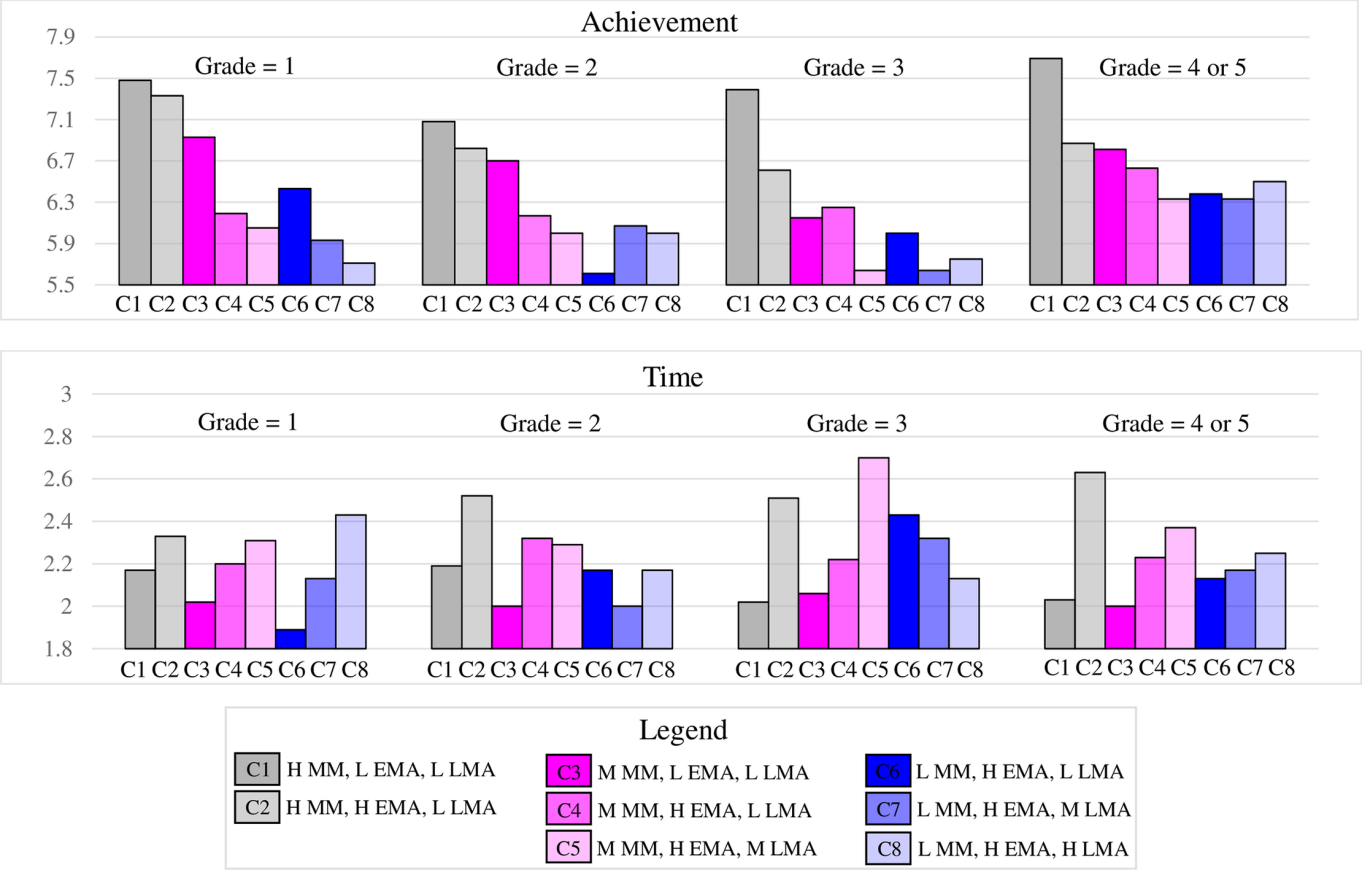
Relations between class memberships and math achievement/math time by grade level. C = class; L = low, M = medium, H = high, MM = math motivation, EMA = exam math anxiety, LMA = learning math anxiety.

Finally, given that the current sample contains students from different grades (year 1 through year 5 of high school), we also explored whether the differences among classes in math achievement and math time varied across grade levels. Table 7 presents the sample size for each class by grade level combination. Given that the present design was cross-sectional, this set of analyses were limited to description and pattern visualization. Fig 3 presents the mean of math achievement (left panel) and math time (right panel) in each class by grade level.

**Table 7:** Sample size for class membership by grade level.

	Class 1	Class 2	Class 3	Class 4	Class 5	Class 6	Class 7	Class 8	Total
Grade = 1	33	46	30	70	39	14	15	7	254
Grade = 2	24	50	37	58	34	18	15	9	245
Grade = 3	32	44	27	48	22	7	14	4	197
Grade = 4/5	29	38	27	62	27	8	24	14	229
Total	117	178	121	238	122	47	68	34	925

Note. Grade level information was missing for 2 individuals.

#### Differences in math achievement among classes

Comparing classes within each MM group allowed us to examine differences in achievement associated with differences in MA. In high and medium MM groups, classes with lower EMA generally appeared to have better performance (i.e., comparing Class 1 against Class 2, and comparing Class 3 against Class 4 and Class 5). There seemed to be little differences in achievement associated with differences in learning MA. Additionally, the differences between Class 1 and Class 2 seemed larger in higher grades, whereas the differences between Class 3 and Class 4/Class 5 seemed smaller in higher grades, suggesting that the negative association between EMA and math achievement may be stronger in highly motivated students. The association pattern between MA and achievement was less clear in the low MM group, potentially due to the small sample sizes in these classes.

#### Differences in math time among classes

Similar to the grade-aggregated pattern, Class 1 and Class 3, the two classes with the lowest overall MA within their respective MM groups, consistently reported relatively low amount of time spent on learning math after school. To the contrary, Class 2 and Class 5, the two classes with the highest overall MA within their respective MM groups, consistently spent relatively more time on learning math after school compared to all other classes. These together suggest that it is the combination of high MM and high MA that drives students to work more on math after class.

## Discussion

The diverse range of emotions and motivations developed during the math learning process has profound implications in mathematics education, as these influence not only the mobilization of cognitive resources during a math test, but also long-term learning behaviors [6, 27–28]. The goal of the current study was to explore differential profiles of the multi-dimensional emotion and motivation factors, and ultimately to investigate how these factors interacted and related to math learning practices and achievement.

### Are highly math anxious students always unmotivated in math?

Using latent profile analysis, we discovered 8 distinct classes capturing various combinations of MA and MM. We further grouped these 8 classes into high, medium, and low MM groups to facilitate the understanding of each class characteristics and comparisons across classes. Contrary to the current understanding in the extant literature that math anxious students generally have low motivation [6, 27], our analyses suggest that some math anxious students are highly motivated. In particular, students in Class 2 are highly motivated, and they also reported high exam MA. Class 4 and 5 reported average to above average MM despite their high levels of exam MA. Therefore, high exam MA seems to be present in students with all levels of motivation.

With respect to learning MA, medium learning MA was observed only in medium and low, but not high, MM groups, and high learning MA was observed only in low MM but not medium or high MM groups, suggesting that higher learning MA was generally associated with lower MM. In summary, highly motivated students are still likely to experience exam MA, but they are less likely to experience learning MA. It is not surprising that students who feel unease in math classes do not enjoy their experience, and this is consistent with the observation that high learning MA was observed only in concomitance with low MM. However, anxiety about an upcoming math exam may reflect students’ lack of confidence in their math abilities, or it may reflect their desire for better achievement, and this is consistent with the observation that high exam MA was observed at all levels of MM. These findings point to the heterogeneous nature of the relations between different aspects of MA and MM [8].

Class memberships differed between genders, such that females were more likely to belong to classes characterized by a combination of lower MM and higher MA compared to males. This is consistent with existing literature on sex differences in math-specific emotions and motivation [6, 29–30].

### How do the dimensions of MM and MA relate to math achievement?

In general, Class 1 reported the highest achievement, followed by Classes 2 and 3. The remaining five classes reported similar achievement, which was lower than that of the first three classes. The general pattern suggests that a combination of higher MM and lower MA is associated with higher achievement, a finding consistent with studies that examined the effects of MA and MM separately [4, 17, 19]. However, this finding seems to be inconsistent with a previous study showing that students with a combination of high MM and medium MA had the highest math achievement [5]. This difference in finding could be attributable to several factors. First, the sample characteristics between the two studies are different: the current study used a sample of high school students from Italy whereas the previous study used samples of American middle school and college students. Second, whereas the previous study focused on the interaction between global MA and global MM, the present study focused on examining specific dimensions of MA and MM. Third, the two studies differ critically on how motivation was measured. The present study focused on aspects of motivation that were widely studied in the literature, including perceived importance of math, self-perceived ability in math, and interest in math. In the previous study, motivation was measured more broadly, encompassing dimensions such as focused attention. Lastly, different measures were used to assess math performance. The previous study relied on lab tasks, whereas the present study used a high-stake exam. It is possible that the relation between moderate levels of MA and math performance further depends on the nature of the math task (i.e., whether the task is high-stake or not) [27]. Future studies should explore which of the above differences contributed to the discrepancy in findings between these two studies.

When the classes were further broken down into different grade levels, specific interaction patterns emerged. Specifically, when comparing classes within each of the high and medium MM groups, (i.e., Class 1 vs. 2, and Class 3 vs. 4), the classes with higher exam MA had poorer math achievement. Additionally, the differences between Classes 1 and 2 appeared larger whereas the differences between Classes 3 and 4 appeared smaller in higher grade levels. This indicates that high exam MA is negatively associated with math performance more strongly in individuals with higher MM, and such effects seem to be stronger in higher grade levels. This result echoes a recent study on math learning, which found that higher stress during learning predicts more forgetting of course content and avoidant thinking about the course only in students with strong math self-concept (i.e., students who believe that they are good at math and that it is important for them to be good at math), but not in those with weak math self-concept [31]. Both sets of results provide support for the identity threat account which argues that domain-specific stress and anxiety impact more heavily on individuals who are motivated in and identify themselves with that particular domain.

Within the low MM group, there seems to be no clear association between exam MA and math achievement. It is possible that low exam MA does not contribute above and beyond the negative association between low MM and poor math achievement. It is also possible that we failed to observe any systematic pattern due to the small sample sizes in the low MM group. Finally, there seemed to be little differences in math achievement associated with differences in learning MA.

### How do the dimensions of MM and MA relate to math avoidance?

Contrary to the current belief that highly anxious students are avoidant of math [6, 27], our findings showed that within each MM group, the more anxious students were generally more engaged across all grade levels. When combined with high motivation, worries about math result in more efforts and investment in math learning rather than more avoidance. The discrepancy between the present finding and previous literature may be attributable to the difference in the operationalization of math avoidance. Most studies to date focused on avoidance behaviors in distal situations where students have more freedom to choose among alternatives [27], such as whether or not to select an elective math course or to take a math-related career in the future. In the present study, we examined how much time high school students spent on learning math after school by taking out-of-school math lessons and studying for math as part of their homework on their own. These activities represent math avoidance behaviors in imminent learning situations in high school when immediate negative consequences ensue from poor performance on required math courses (e.g., low GPA negatively influence college application).

Previous literature on threat response suggests that threat can induce both approach-oriented and withdrawal-oriented responses depending on the characteristics of the threatening situation. An escapable threat of greater distance more likely elicits withdrawal/avoidance behaviors, whereas an inescapable threat in close distance more likely elicits approach-oriented responses [32]. Thus, it is possible that high MA students rely on different strategies to deal with their negative emotions in different learning situations: they invest more effort in math learning to avoid the immediate negative consequences associated with poor math performance, but they choose to disengage from math learning when it yields no immediate negative outcome.

It is worth noting that within each MM group, although classes with higher MA reported more learning efforts, they generally had lower achievement compared to classes with lower MA. In other words, within each MM level, high MA students worked longer hours but performed worse in math. Two possible mechanisms may explain this counterintuitive finding. Previous studies have shown that MA hinders math performance through compromising working memory efficiency [16], a cognitive ability that plays an important role in both math performance and math learning [33–34]. For students who reported MA on exam but not learning (e.g., Class 2), MA may interfere only with performance on high stake math exams [27] but not knowledge acquisition in math lessons. Therefore, high stake exam performance may not reflect students’ true math abilities in those with high exam MA [27]. Because students with high exam MA consistently expend extra effort in learning math, it is possible that they master more knowledge than what is reflected through their exam performance, but such knowledge can only be induced through alternative low-stress assessments such as untimed testing. However, the mechanism may be different for students who suffer learning MA. For these students, impairments in math cognition may be more profound as MA may also hinder the knowledge acquisition process in daily math lessons. As such, these students may need to study for longer hours than their peers to complete the same amount of work because of their poorer math abilities.

The present study has some limitations. First, all main constructs were self-reports. As a result, relations among variables may have been inflated due to method artifact. A multi-informant multi-method design is needed to test the replicability and generalizability of the current results. Second, two of the scales (importance and math time) had too few items to assess their internal reliability. However, their correlations with other variables were consistent with the existing literature, providing evidence supporting their predictive validity. Finally, because of the cross-sectional design, we were not able to statistically examine how the differences among the eight classes changed over time. Future longitudinal data from the MILES project will allow us to better understand the developmental trajectories of these diverse profiles.

In summary, the present study explored profiles of math-specific emotions and motivations in adolescence. Our findings revealed that the relation between MA and MM and their combined roles in math achievement and math avoidance are complex. These diverse profiles call for customized interventions to address the heterogeneous mechanisms underlying low math achievement. For some students, it may be sufficient to address how anxiety affects online cognitive processing during high-stake math exams to ensure that the educational assessment reflects their true abilities. For other students, it is more pressing to address how anxiety interferes with attention and memory processes that are needed for optimal learning in daily math lessons. Finally, for the least motivated students, building the internal drive for mathematics may be a priority.
